# SPME-GC/MS Analysis of Methanol in Biospecimen by Derivatization with Pyran Compound

**DOI:** 10.3390/molecules25010041

**Published:** 2019-12-20

**Authors:** Joon-Bae Lee, Yong Ae Jeong, Dae Jun Ahn, Iel Soo Bang

**Affiliations:** 1 Daejeon Institute, National Forensic Service, Daejeon 34054, Korea; jya79421@gmail.com; 2 Gwangju Institute, National Forensic Service, Jeonnam 57248, Korea; djahn7@korea.kr; 3 Department of Microbiology and Immunology, Chosun University School of Dentistry, Gwangju 61452, Korea

**Keywords:** methanol, derivatization, solid-phase microextraction, gas chromatography/mass spectrometry

## Abstract

Methanol is metabolized in the body to highly toxic formaldehyde and formate when consumed accidentally. Methanol has been typically analyzed with gas chromatography-flame ionization detector (GC-FID). However, its retention time may overlap with other volatile compounds and lead to confusion. Alternative analysis of methanol using gas chromatography/mass spectrometry (GC/MS) also has limitations due to its similar molecular weight with oxygen and low boiling point. In this study, methanol and internal standard of deuterium-substituted ethanol were derivatized with 3,4-dihydro-2H-pyran under acid catalysis using concentrated hydrochloric acid. The reaction products including 2-methoxytetrahydropyran were extracted with solid-phase microextraction followed by GC/MS analysis. This method was successfully applied to measure the lethal concentration of methanol in the blood of a victim with a standard addition method to overcome the complex matrix effect of the biospecimen. Identification of the metabolite formate by ion chromatography confirmed the death cause to be methanol poisoning. This new method was a much more convenient and reliable process to measure methanol in complex matrix samples by reducing sample pretreatment effort and cost.

## 1. Introduction

Methanol is a highly versatile chemical used in the manufacture of solvents, pharmaceuticals, pesticides, fuels, resins, and electronics. It is easily accessible from our surroundings, but accidental or intentional ingestion can lead to life-threatening health conditions [1]. Methanol, when absorbed into the body, is metabolized to produce highly toxic formaldehyde and formate [2], which can cause blindness and death. Methanol is converted to formaldehyde by alcohol degrading enzyme (ADH) and to formate by aldehyde degrading enzyme (ALDH) in the liver [3]. The toxic effects of formate can damage the optic nerves and prevent the energy metabolism of the mitochondria, which eventually destroys the living body [4]. The amount of poisoning and lethal dose of methanol varies between individuals, but generally, the acute ingestion of 10 mL of methanol can cause permanent blindness and 100–200 mL can be fatal to most adults [5]. It was reported that the blood methanol levels from 504 to 1286 μg/mL through inhalation exposure resulted in altered vision [6]. For the fatal cases by methanol intoxication, the concentration in blood showed a wide range from 150 to 4820 μg/mL [7]. Methanol poisoning may be caused by the consumption of bootleg liquor and pesticides, misuse of medicines, or intake of chemicals containing methanol for suicide. First-aid measures include gastric lavage and administration of ethanol to slow down the metabolism of methanol [8,9]. Therefore, in the case of methanol poisoning, ethanol administration is an important method of first aid [10]. Methanol is generally analyzed by a gas chromatography-flame ionization detector (GC-FID) [11,12]. However, this method has some weakness due to low molecular weight and low boiling point (65 °C) of methanol which causes retention time overlap with other volatile organic compounds appearing at the beginning of the analysis [13]. Moreover, in the case of complex matrix samples, such as blood, the analytes with early retention time could be confused as a methanol peak. For this reason, the GC-FID, which depends on only retention time, could be difficult for qualitative and quantitative analysis of methanol in complex matrix samples. In order to overcome this drawback, analysis using the gas chromatography-mass spectrometry (GC/MS) could be a possible alternative. However, the molecular weight of methanol is similar to that of oxygen, and column bleeding may occur due to moisture from the injected volume of the headspace in the sample [14]. To solve the flaw of GC/MS in this study, methanol was derivatized in the sample and analyzed using GC/MS. Conventional pretreatment techniques for instrumental analysis include solvent extraction methods such as liquid-liquid extraction [15] and solid-phase extraction [16]. These processes not only require large amounts of solvents with high purity but also consume lots of time and effort resulting in high analysis costs. Consequently, derivatization reagents were added to the vial-containing sample, reaction products were extracted with solid-phase micro extraction (SPME) and directly analyzed by GC/MS [17,18,19]. In order to rule out external influences, deuterium-substituted ethanol (C_2_D_5_OH) was used as an internal standard (IS) and standard addition method was employed to avoid the matrix effect of the blood sample on the quantification of methanol [20]. Derivatization of methanol and IS was carried out with 3,4-dihydro-2*H*-pyran (DHP), a pyran-based compound under acidic conditions, and extraction was carried out using SPME fiber of carboxen/PDMS (CAR/PDMS) which possesses the best analytical properties among commercial SPME fibers [21,22]. Moreover, this study investigated the cause of death in a victim who consumed veterinary skin-care medicine by analyzing the metabolite, formate, as well as methanol levels in the blood. 

## 2. Results and Discussion

### 2.1. GC-FID Analysis

**Figure 1:**
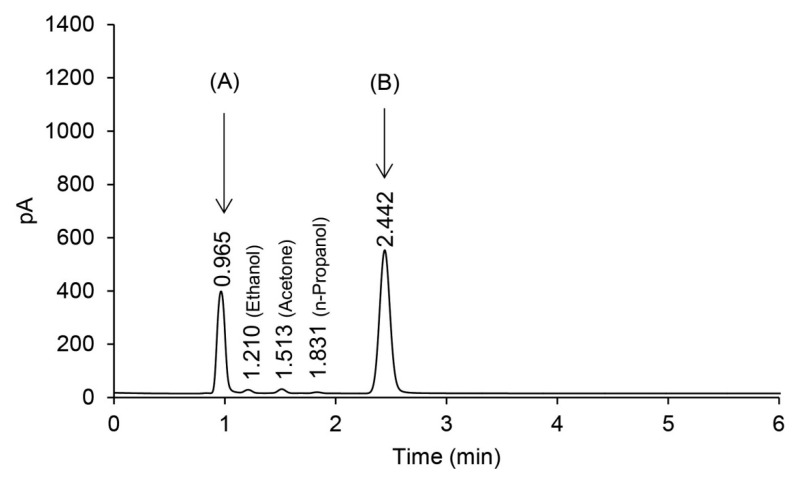
Gas chromatography-flame ionization detector (GC-FID) chromatogram for methanol or acetaldehyde (**A**) and internal standard (t-butyl alcohol) (**B**).

Blood methanol was analyzed using GC-FID. As shown in Figure 1, the result shows a peak retention time of 0.96 min supposed to be for methanol and 2.4 min for t-butyl alcohol. However, it was difficult to determine if the peak at 0.96 min exactly corresponds to methanol by only retention time. This is because the peak at 0.96 min could be derived from methanol, or from acetaldehyde, a metabolite of ethanol because when methanol is consumed with drinking alcoholic beverages, both compounds could appear at the same retention time and, therefore, lead to confusion. 

### 2.2. Derivatization and GC/MS Analysis

**Figure 2:**
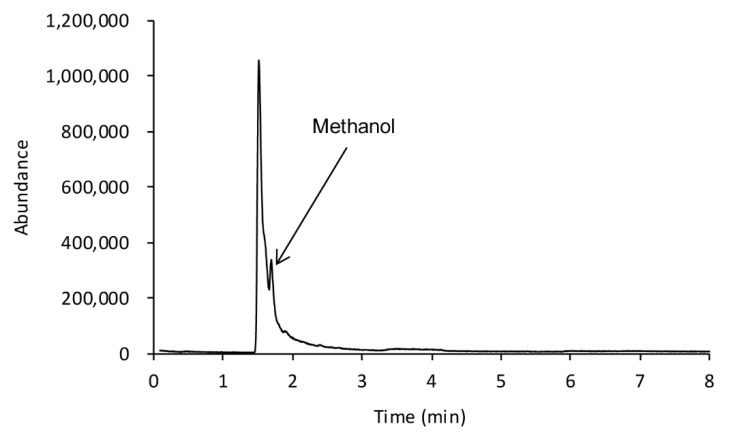
Gas chromatography/mass spectrometry (GC/MS) chromatogram for methanol in the blood sample.

Analysis using gas chromatography-mass spectrometry (GC/MS) enables the identification of chemicals through the interpretation of the mass spectrum. In this analysis, methanol appears very quickly (Figure 2) and could be overlapped with other compounds due to its low molecular weight which is similar to oxygen. To prevent this, methanol was analyzed using GC/MS through derivatization. 

Derivatization of methanol was carried out using a pyran-based compound, 3,4-dihydro-2*H*-pyran (DHP), also known for protecting alcohol groups in organic synthesis. The reaction proceeds easily and quickly and its product is stable enough to be commercially traded.

The internal standard (IS) used for quantification was ethanol substituted with deuterium (C_2_D_5_OH). Deuterium-substituted ethanol is extremely unlikely to exist in the natural environment. Owing to this property, it could be applied to the quantitative analysis of ethanol as an internal standard by GC/MS analysis, particularly in the drinker’s blood.

**Figure 3:**
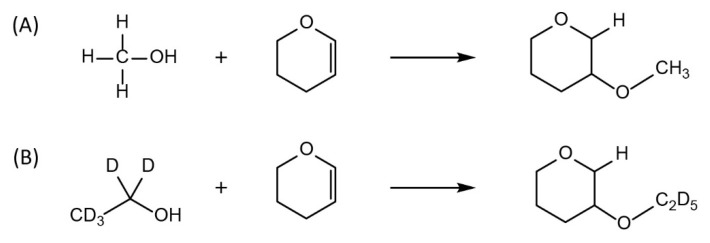
Reaction schemes of methanol (**A**) and deuterium substituted ethanol (**B**) with 3,4-Dihydro-2H-pyran (DHP).

Derivatization of methanol and IS was easily performed under acidic conditions, especially with concentrated hydrochloric acid, and it showed reliable results. Under the acidic condition, the reaction of DHP with methanol and IS proceeded through nucleophilic addition, which gave 2-methoxytetrahydropyran and 2-d5-ethoxytetrahydropyran, respectively, as shown in Figure 3.

**Figure 4:**
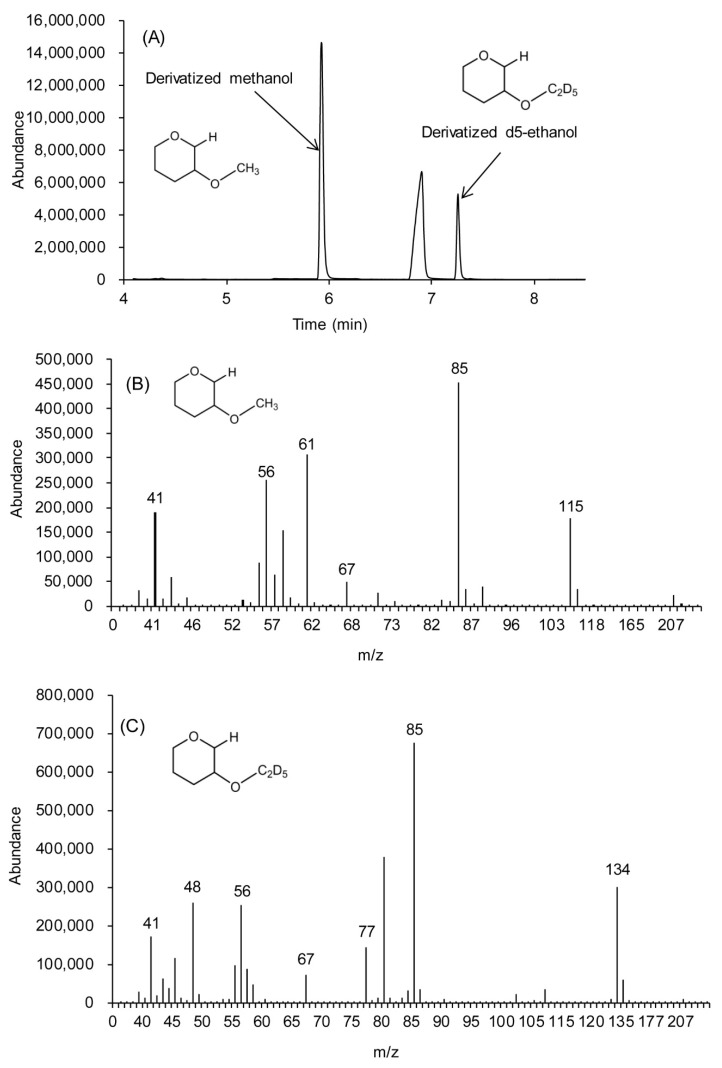
GC/MS chromatogram of derivatized methanol in blood sample (**A**), mass spectra of derivatized methanol (2-methoxytetrahydropyran) (**B**) and internal standard (2-d5-ethoxytetrahydropyran) (**C**).

The derivatized methanol and IS appeared at the retention time of 5.9 and 7.3 min, respectively, at the given GC/MS condition. GC/MS chromatogram and the mass spectra for each component are shown in Figure 4A–C. Especially, the peak at the retention time of 6.9 min in Figure 4A seems to be from the fragmented DHP or derivatized compounds, which has the *m*/*z* of 101 of molecular ion and *m*/*z* 51 of base ion and is supposed to be from butyl formate.

**Figure 5:**
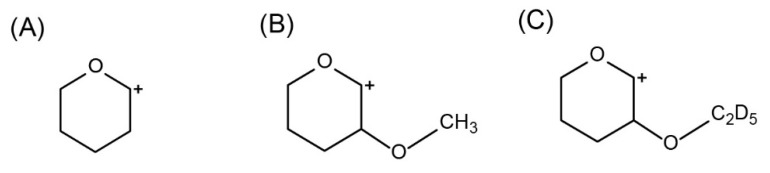
The proposed structure for the ion with *m*/*z* 85 for both derivatives (**A**), molecular ions with *m*/*z* 115 for derivatized methanol (**B**) and *m*/*z* 134 for derivatized internal standard (**C**).

The base ion for both derivatized products was an ion with *m*/*z* 85 altogether. Molecular ion for derivatized methanol and IS was an ion with *m*/*z* 115 (Figure 4B) and *m*/*z* 134 (Figure 4C), respectively. The proposed structure for the base ion is shown in Figure 5A and quantitative ions were shown in Figure 5B,C.

**Figure 6:**
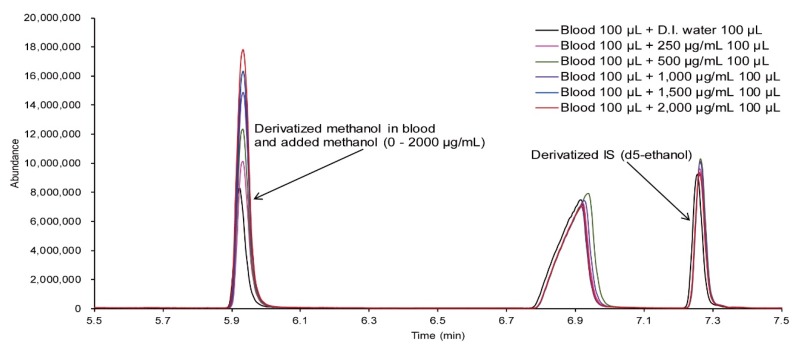
GC/MS chromatograms for the blood of the victim with standard methanol solution.

**Figure 7:**
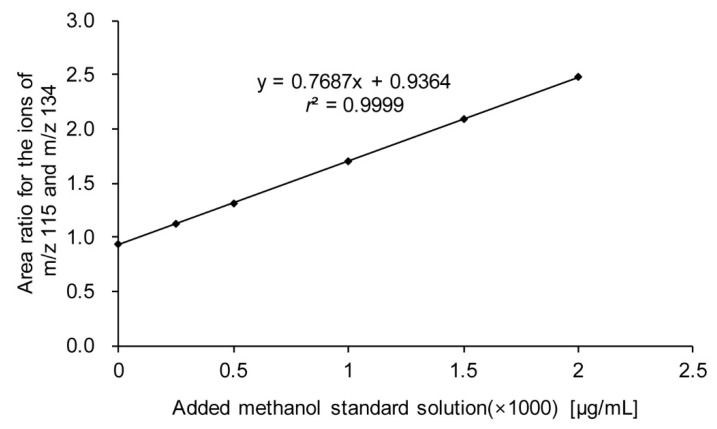
Calibration curve derived by plotting the area ratio of the ion with *m*/*z* 115 (derivatized methanol) and the ion with *m*/*z* 134 (derivatized internal standard) against the concentration.

The results obtained using GC/MS analysis by standard addition of methanol within the range of 0–2000 μg/mL to the blood of the victim are shown in Figure 6. The calibration curve was prepared by using the area ratio of the ion with *m*/*z* 115 for methanol and the ion with *m*/*z* 134 for IS, as shown in Figure 7. The concentration of methanol in the blood of the victim was found to be 1218 μg/mL by calculating the slope and intercept of the curve.

For the linearity of the calibration curve, the *r*^2^ value was 0.9999, the detection limit was 0.56 μg/mL, and the quantification limit was 1.7 μg/mL. For the detection limit and quantification limit, S/N ratios of 10 blank samples were calculated with concentrations exceeding 3 and 10.

### 2.3. Extraction

For the GC/MS analysis of the 2-methoxytetrahydropyran from the reaction of methanol and DHP, extraction was performed using SPME (solid-phase micro extraction) at the headspace of the sample vial.

**Figure 8:**
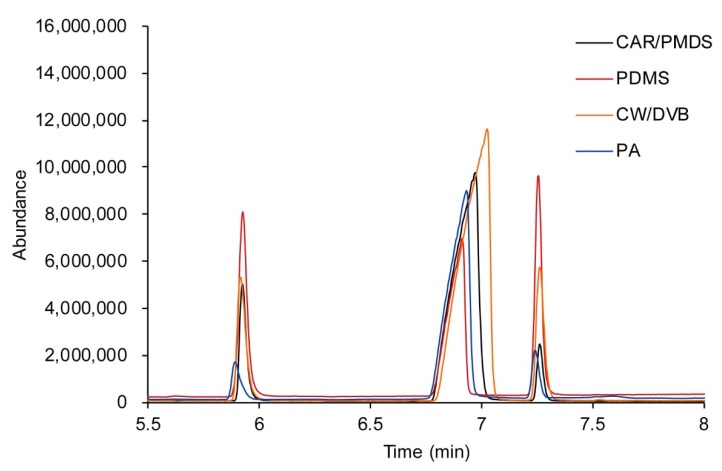
GC/MS chromatograms for the comparison of extraction characteristics of derivatized methanol and the internal standard by using commercial solid-phase micro extraction (SPME) fibers.

Characteristics of commercial SPME fibers such as carboxen/polydimethylsiloxane (CAR/PDMS), polyacrylate (PA), PDMS, and carbowax/divinylbenzene (CW/DVB) were compared with one another for each sample containing methanol of 1000 μg/mL and internal standard. The results are shown in Figure 8.

**Figure 9:**
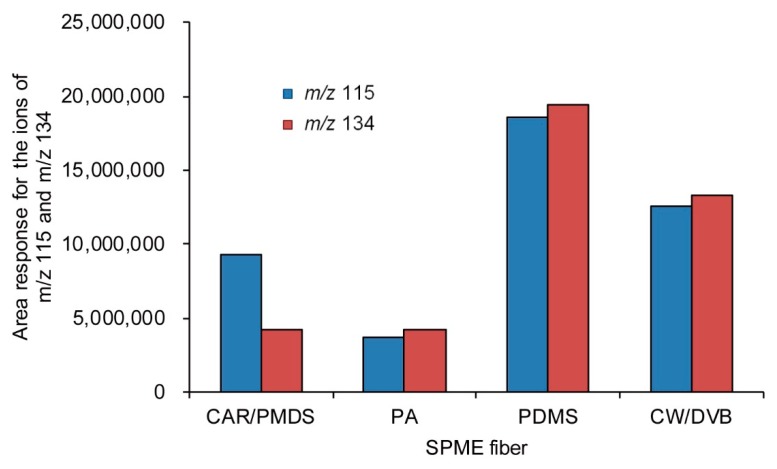
Comparison of the area response for the ion of *m*/*z* 115 (for the derivatized methanol) and the ion of *m*/*z* 134 (for the derivatized internal standard) by using CAR/PDMS, PA, PDMS, and CW/DVB SPME fibers.

When comparing the area ratio of the ion with *m*/*z* 115 and that of *m*/*z* 134, the fiber composed of CAR/PDMS showed better area response for the derivatized methanol than that of the derivatized internal standard and was used for this analysis (Figure 9).

### 2.4. IC Analysis

Blood diluted with 50 parts of demineralized water was analyzed using IC (ion chromatography) to screen the metabolite of methanol poisoning.

**Figure 10:**
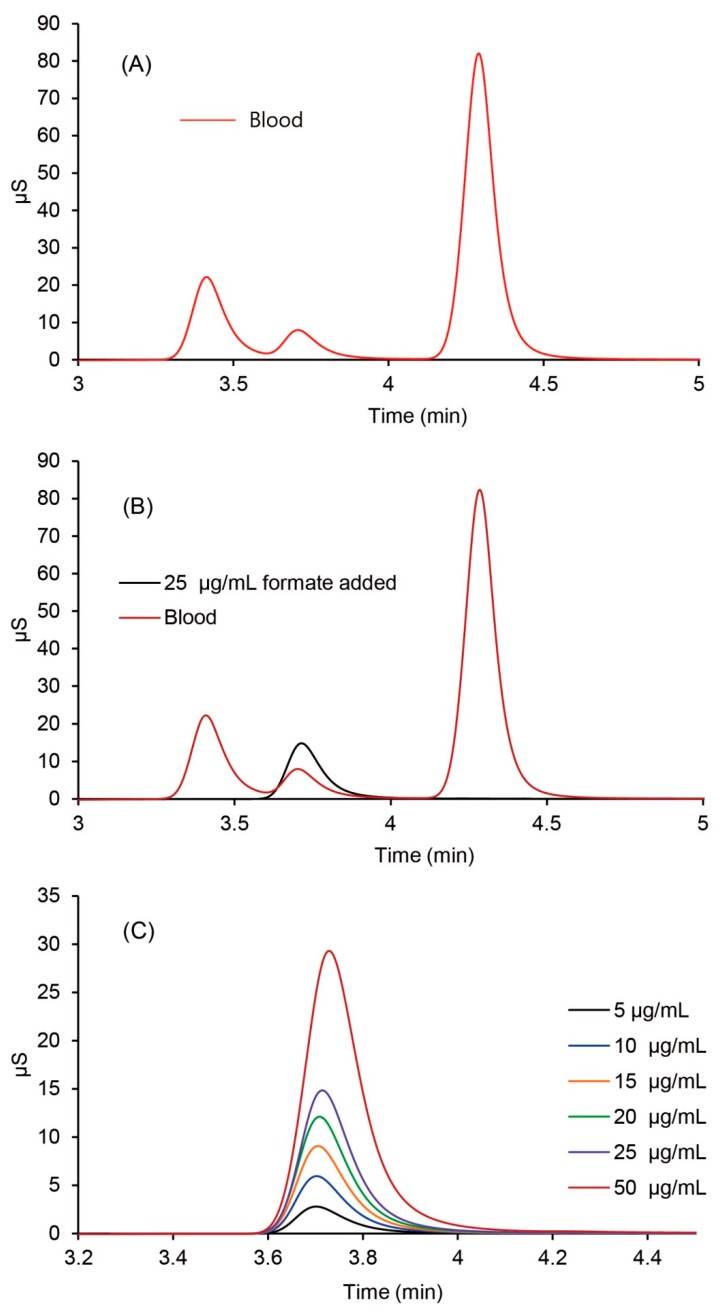
Ion chromatography chromatograms for the diluted blood sample of the victim (**A**), standard formate ion added to the blood sample (**B**), and formate ion standards (**C**).

In Figure 10A, a peak was detected at the retention time of 3.7 min, which was from the formate ion. To confirm the peak, the same volume of a standard formate solution (25 μg/mL) as the diluted blood sample was added to the sample followed by IC analysis. The result showed identical peaks without splitting, and hence it was confirmed to be from the formate ion, as shown in Figure 10B.

**Figure 11:**
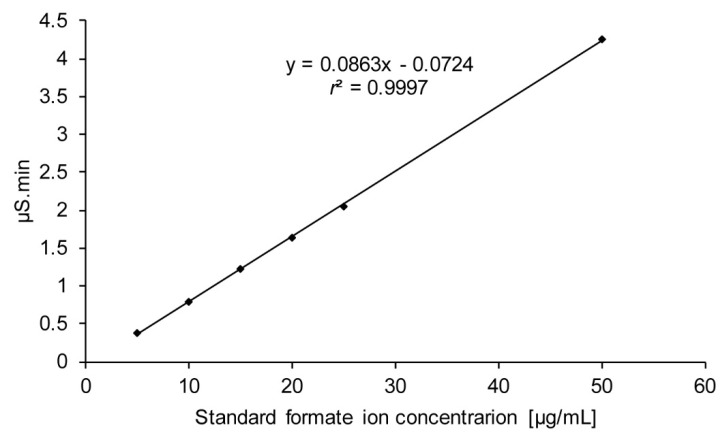
Calibration curve of the formate ion against the concentration of 5–50 μg/mL of formate ion using ion chromatography.

For the quantification of formate, a calibration curve was constructed by plotting the area of formate ion against the concentration of 5–50 μg/mL (Figure 10C), with an *r*^2^ value of 0.9997 showed good linearity (Figure 11). Moreover, 590 μg/mL of formate ion was measured in the blood sample by this calibration curve.

## 3. Materials and Methods

### 3.1. Chemicals and Reagents

Methanol of analytical standard grade and SPME fibers were purchased from Supelco (Bellefonte, PA, USA) The fibers of carboxen/polydimethylsiloxane (CAR/PDMS), polydimethylsiloxane (PDMS), polyacrylate (PA) and carbowax/divinylbenzene (CW/DVB) were tested for the characteristics of methanol analysis. 3,4-Dihydro-2*H*-pyran (DHP) used for derivatization was purchased from TCI (Kita-ku, Tokyo, JPN) and ethanol-1,1,2,2,2-d5 from Sigma-Aldrich (Darmstadt, Hessen, GER). For IC analysis anion eluent used was composed of carbonate/bicarbonate from Thermo Fisher Scientific (Sunnyvale, CA, USA) and used after 100-fold dilution. Formate ion standard solution was purchased from AccuStandard (New Haven, CT, USA).

### 3.2. Instruments

Methanol analysis was performed using GC-FID with 6890 GC and 7697A Autosampler from Agilent Technologies (Foster City, CA, USA) equipped with the column of 80/100 0.20 ‰ CW-1500 Carbopack C 6 ft × 1/8 in × 2.1 mm SS from Supelco (Bellefonte, PA, USA). The temperature of the autosampler oven and the transfer line was 65 °C and 120 °C, respectively. The time for vial equilibration was 5 min, injection duration 1 min, and cycle 4.5 min. The carrier gas used was 99.9999% He at a flow rate of 13.9 mL/min. GC inlet temperature was 200 °C, and carrier gas was 99.9999% He with a flow rate of 14.7 mL/min. GC oven temperature was maintained at an isothermal state of 120 °C with a run time of 3.5 min. For FID detector, the flow rate of hydrogen and air was 40 mL/min and 350 mL/min, respectively, and temperature was maintained at 250 °C. GC/MS analysis was performed using the 7890B GC and 5977B MSD system from Agilent Technologies (Foster City, CA, USA) equipped with DB-5MS UI capillary column (30 m length × 0.25 mm id, 0.25 μm film thickness, J & W Scientific, Folsom, CA, USA) and carrier gas was 99.9999% He with flow rate of 1.0 mL/min at constant flow mode. The temperature of injector and interface was set to 260 °C and 280 °C. The GC oven temperature was at 40 °C for 3 min, and then heated to 250 °C at the rate of 10 °C/min and then maintained for 3 min. The solvent delay time was set to 4.2 min to exclude the effect of the derivatization reagent. The split ratio of the inlet was 10:1, and analysis by mass spectrometer was performed in EI mode of 70 eV. IC analysis was performed by injecting 5 μL of the sample to the ICS 5000 system of Dionex ((part of Thermo Scientific) Sunnyvale, CA, USA). In order to prevent column contamination, IonPac AG-11 4 mm guard column from Thermo Scientific (Sunnyvale, CA, USA) was used and separated into IonPac AS-11 4 mm column. For eluent, carbonate/bicarbonate eluent was diluted to 4.5/1.5 mM concentration and analyzed with a conductivity detector by applying a current of 26 mA to Thermo Scientific’s ASRS 4 mm suppressor at 1 mL/min of isocratic flow rate. 

### 3.3. Sample Preparation

**Figure 12:**
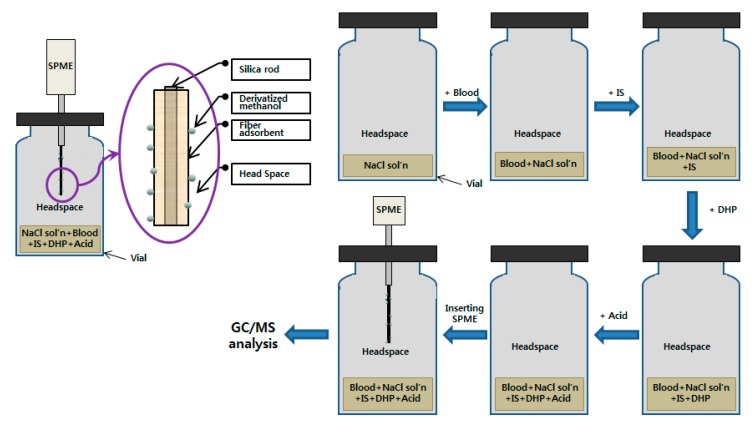
General procedure for the GC/MS analysis of methanol in blood samples through derivatization with DHP using SPME.

The GC-FID analysis of methanol was performed with 200 μL of blood in a 10 mL glass vial containing 200 μL of saturated aqueous sodium chloride (NaCl) solution and 100 μL of 0.05% t-butyl alcohol aqueous solution (internal standard). The homogenized sample was analyzed by warming at 65 °C for 5 min in the oven of an autosampler and then injecting 500 μL of the headspace of the sample vial into the GC inlet. GC/MS analysis for the blood was performed by the standard addition method. For GC/MS analysis with derivatization, 100 μL of blood sample and 100 μL of 0.1% D5-EtOH aqueous solution (internal standard) were added sequentially into a 10 mL glass vial containing 200 uL of saturated aqueous NaCl solution, then 100 μL of methanol standard solution of varying concentrations (250 μg/mL, 500 μg/mL, 1000 μg/mL, 1500 μg/mL, and 2000 μg/mL) were added to each vial followed by a 35 μL of derivatizing reagent (DHP). Then 25 μL of concentrated hydrochloric acid was added to the homogenized vial to derivatize. The 2-methoxytetrahydropyran at the headspace was extracted using SPME. Extraction was subjected to exposure for 10 sec using 75 μm CAR/PDMS SPME fiber and then directly analyzed using GC/MS (Figure 12).

## 4. Conclusions

In this study, GC/MS analysis coupled with SPME through derivatization using 3,4-Dihydro-2*H*-pyran (DHP) as a derivatizing reagent and ethanol substituted with deuterium as an internal standard produced successful results in analyzing methanol. This analysis appeared to be a convenient, cheap, and reliable method compared to the conventional analysis using GC-FID or GC/MS without derivatization. When applied to the forensic case, 1218 μg/mL methanol was measured in the blood of the victim, and formate ion was found to be 590 μg/mL by ion chromatography, which indicated the cause of the death to be methanol poisoning. Based on the outcome of this method, it could be applied reliably to measure other kinds of alcohol existing in complex matrix compounds.
